# How far are we from understanding the genetic basis of Hashimoto’s thyroiditis?

**DOI:** 10.1186/1756-6614-6-S2-A21

**Published:** 2013-04-05

**Authors:** Alicja Hubalewska-Dydejczyk

**Affiliations:** 1 Department of Endocrinology, Jagiellonian University Medical College, Krakow, Poland

## 

Hashimoto’s thyroiditis (HT) is a common autoimmune disease affecting the thyroid. The prevalence of the disease has been increasing in the last years and in many cases it clusters in families.

It is currently believed that susceptibility to HT involves interaction between mutation(s) in generally autoimmune-predisposing genes HLA-DR, PTPN22, CTLA-4, and thyroid autoantigen-specific genes, as well as environmental factors.

Several mutations have been associated with HT susceptibility, including mutations predisposing to autoimmune disorders in general. The most often cited include PTPN22 c.1858C>T (p.R620W), and CTLA4 c.49A>G (p.T17A) and CT60 (c.6230A>G) in the 3’UTR region. Polymorphisms in CTLA4 contribute to a higher production of autoantibodies in HT patients. In addition, the protective A allele at position 49 causes an increased amount of soluble form of CTLA4, which is much lower in CT60 G/G-positive disease-susceptible individuals. Contrary to expectations, the PTPN22 c.1858C>T transition has been shown to be a gain-of-function mutation, which inhibits TCR signalling. So far only the thyroid-specific gene associated with HT, thyroglobulin (TG), the exons 10 -12 SNP cluster (p.S734A, p.P778=, p.M1027V) has been found to be more prevalent in HT patients. Homozygosity for the C susceptibility allele of an exon 33 SNP (p.R1999W), together with the presence of 1 or 2 copies of the susceptibility allele of one of the 10-12 exons SNP cluster seems to confer the strongest association with AITD for this gene.

However, none of the mentioned genes has an effect which would be large enough to explain the predisposition to HT on its own. It is currently accepted that it is rather interaction of a couple of altered genes rather than one single gene, which triggers the phenotype of disorder. In contrast to many other autoimmune diseases, it was impossible to unanimously associate HT with one HLA-DR type. It has but been found that a specific amino acid signature in exon 2 of the HLA-DRB1 gene is important for HT susceptibility. Additional presence of the exon 33 SNP in the TG gene causes a synergistic effect on disease susceptibility. This co-existence probably causes the preferred presentation of mutated TG to autoreactive T-cells by the altered HLA pocket, which then lead to AITD development.

Other mutations could partially explain the impact of environmental factors on HT susceptibility: Carriers of the susceptibility allele at position -1623 of the TG promoter region are prone to produce high amounts of TG when encountering IFNα.

In summary however, it is impossible to identify a single etiological polymorphism or even gene, which would be responsible for the etiopathology of Hashimoto’s thyroiditis. It seems that there are at least a couple of genes responsible for this disease and that different mutations could be responsible for the different phenotypes of this disorder like the age of symptom onset, occurrence of postpartum thyroiditis, presence of goitre, or a coexisting thyroid carcinoma.

